# Coverage disparities in mobile health services for migrant workers in Korea: a spatial equity analysis

**DOI:** 10.7189/jogh.15.04300

**Published:** 2025-10-17

**Authors:** Kyoung Kyun Oh, Yunnam Kim, Wonseok Park, Joohwan Cheon

**Affiliations:** Department of International Cooperation, Korea Foundation for International Healthcare, Seoul, Republic of Korea

## Abstract

**Background:**

Migrant workers in the Republic of Korea face substantial barriers to healthcare due to linguistic, legal, and financial constraints, particularly among low-skilled labourers in high-risk occupations. Since 2008, the Korea Foundation for International Healthcare has operated a mobile clinic programme to deliver essential medical services to migrant workers. Despite its critical role, no systematic evaluation has been conducted to date. We examined the design and implementation of the 2024 programme, with a focus on regional disparities and health equity.

**Methods:**

We conducted a cross-sectional descriptive analysis of mobile clinic operations from May 2024 to March 2025 across 22 city- and district-level sites. We calculated coverage proportion as the number of clinic beneficiaries divided by the registered E-type visa holder population. We visualised spatial disparities using graduated symbol maps and analysed regional differences using the Kruskal-Wallis test, followed by Dunn’s test with Bonferroni correction.

**Results:**

A total of 2336 migrant workers received services through 50 deployments, most of whom were male from Southeast and South Asia. Internal medicine (47.9%), general services (21.1%), and traditional Korean medicine (9.1%) were the most delivered services. Coverage ranged from 0.5% to 46.9% across regions. Higher coverage was observed in Seoul districts, while industrial hubs such as Hwaseong and Pyeongtaek showed lower outreach. There were no statistically significant differences in coverage proportions across the five regional clusters (H = 9.470; df = 4; *P* = 0.0503; 95% confidence interval = 4.37–17.51). However, *post-hoc* analysis identified a statistically significant difference between Seoul and Seoul Metropolitan Area excluding Seoul (z = 2.46; *P* = 0.0410; 95% confidence interval = 0.91–4.83). Civil society organisations played a central role in programme implementation.

**Conclusions:**

Mobile clinics provide a scalable, equity-oriented model to enhance the health access of migrant workers. However, addressing geographic disparities requires data-driven site selection and institutionalised collaboration with civil society. Locally adapted mobile clinic strategies are crucial for ensuring equitable access and the sustainable integration of migrant workers into healthcare systems.

**Registration:**

Open Science Framework (https://osf.io/wx7nz/).

Global migration reached 258 million in 2017, representing a nearly 50% increase compared to 2000 and underscoring its growing implications for global public health and policy [1]. Migration is now widely recognised as a key social determinant of health, with migrants frequently encountering systematic barriers that adversely impact their health-related quality of life [2]. Migrants are likely to face limited healthcare access, language and cultural barriers, precarious employment, and social marginalisation, which contribute to their disproportionate burden of musculoskeletal disorders, occupational injuries, mental health challenges, and chronic diseases due to harsh working and living conditions [2–6].

In the Republic of Korea, foreign labour has become economically essential due to population ageing and growing workforce shortages. As of December 2024, approximately 2.65 million foreign nationals resided in Korea, accounting for 5.2% of the total population [7]. Under the Employment Permit System, a significant share are migrant workers engaged in labour-intensive sectors, such as manufacturing, agriculture, and construction, commonly referred to as ‘3D’ (*i.e.* dirty, dangerous, and demeaning) jobs [8,9]. These migrant workers encounter heightened occupational and psychosocial risks, but frequently face substantial linguistic, legal, and financial barriers to accessing quality health services, particularly among those without documentation or adequate insurance coverage [3,10–13]. Undocumented individuals often avoid care due to fears of deportation or discrimination, while many insured workers remain unaware of available health resources or how to navigate the healthcare system [14,15]. High levels of acculturative stress and social isolation can undermine mental health and reduce engagement in preventive health behaviours [16,17].

To address these challenges, Korea Foundation for International Healthcare (KOFIH) has implemented a range of initiatives since 2006, in accordance with the KOFIH Act. Such initiatives include the development of multilingual health information dissemination, expansion of immunisation programmes, and the mobilisation of a mobile clinic programme aimed at improving access to care for medically underserved migrant communities in Korea. The mobile clinic programme, implemented in collaboration with local health authorities and other partners since 2008, provides a range of appropriate services, including primary healthcare, immunisation, health education, and chronic disease screening. The programme is grounded in a commitment to upholding the health rights of migrant workers and fostering a healthy multicultural society.

We aimed to examine the 2024 implementation of the mobile clinic programme, focussing on regional disparities and health equity. By combining routine service data with geospatial analysis, we sought to evaluate how the mobile clinic programme contributes to closing access gaps across Korea’s diverse migrant workers.

## METHODS

### Study design and setting

We employed a cross-sectional, descriptive design based on secondary data collected during the 2024 mobile clinic programme implemented by KOFIH and Kyung Hee University Medical Center. The programme operated between May 2024 and March 2025 across 22 city- and district-level localities in Korea. A total of 50 mobile clinic deployments were conducted during this period. Seoul, the capital city, was further subdivided into administrative districts due to its high density of migrant populations. Civil society organisations (CSOs) selected service areas in consultation with local health authorities, considering administrative requests, assessed needs, and logistical feasibility. As of December 2024, Korea had 1 488 353 registered foreign nationals, comprising approximately 5.2% of the national population. Most migrants resided in Gyeonggi-do (n = 467 398), followed by Seoul (n = 265 544), Gyeongsangnam-do (n = 102 333), Chungcheongnam-do (n = 96 687), and Incheon (n = 89 129). The five largest foreign national groups were Chinese (n = 465 881; 31.3%), Vietnamese (n = 261 581; 17.6%), Nepali (n = 72 151; 4.8%), Uzbek (n = 61 733; 4.1%), and Cambodian (n = 61 006; 4.1%) [7]. These demographic patterns provided important context for targeting mobile clinic operations and assessing service coverage across diverse migrant workers (**Table 1**).

**Table 1:** Regional distribution of registered foreign residents in Korea (as of 31st December 2024) [7]

Region	n (%)
Gyeonggi-do	467 398 (31.4)
Seoul	265 544 (17.8)
Gyeongsangnam-do	102 333 (6.9)
Chungcheongnam-do	96 687 (6.5)
Incheon Metropolitan City	89 129 (6.0)
Gyeongsangbuk-do	76 768 (5.2)
Jeollanam-do	57 189 (3.8)
Chungcheongbuk-do	56 301 (3.8)
Busan Metropolitan City	55 805 (3.8)
Jeollabuk-do	43 795 (2.9)
Daegu Metropolitan City	36 710 (2.5)
Jeju Special Self-Governing Province	27 990 (1.9)
Ulsan Metropolitan City	27 642 (1.9)
Gwangju Metropolitan City	26 557 (1.8)
Daejeon Metropolitan City	26 249 (1.8)
Gangwon-do	26 074 (1.8)
Sejong City	6182 (0.4)

We aimed to describe demographic characteristics of migrant workers based on available data (*e.g.* nationality and sex), assess regional service coverage, and examine the roles and operational contributions of CSOs to mobile clinic implementation.

### Mobile clinic operation

**Figure 1:**
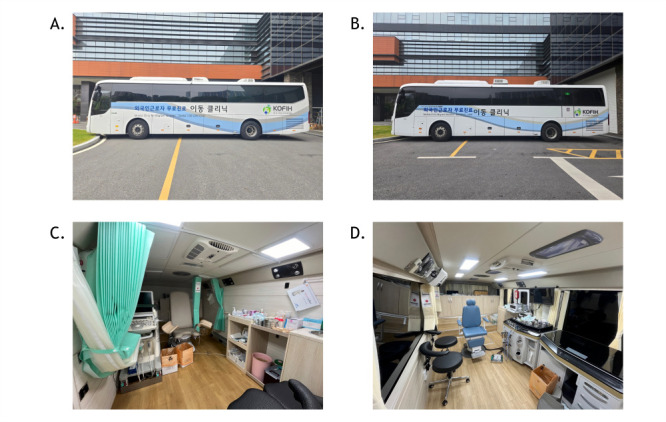
Photographs of two mobile clinic vehicles equipped to provide dental, OBGYN, ophthalmological, and otorhinolaryngological services. **Panel A.** Mobile clinic vehicle 1 (dental & OBGYN). **Panel B.** Mobile clinic vehicle 2 (ophthalmology & otorhinolaryngology). **Panel C.** OBGYN facilities in vehicle 1. **Panel D.** Otorhinolaryngological facilities in vehicle 2. All photographs were taken by authors (Oh K and Kim Y) on 10 May 2025. OBGYN – Obstetrics and Gynaecology.

Two mobile clinic vehicles were deployed, one offering dental and obstetric/gynaecologic services, the other ophthalmology and otorhinolaryngology services (**Figure 1**, Panels A–D). Each vehicle was equipped with diagnostic tools, including electrocardiography, bone densitometry, and an ultrasound machine. Deployments followed a five-step protocol: scheduling, preparation, service delivery, disinfection, and reporting. Requests were submitted through an online platform and reviewed for clinical and logistical feasibility. On-site services were provided in tents and mobile units. Each mobile clinic deployment typically offered daily-based services, serving an average of 40–60 individuals, depending on site conditions, staff availability, and coordinating environment. The clinical team included physicians from multiple specialities, nurses, pharmacists, biomedical engineers, and administrative volunteers, though the exact capacity varied by location. Interpreters and bilingual volunteers offered multilingual support. All reusable medical equipment underwent a three-stage sterilisation process, and medical waste was managed in accordance with national regulations. Monthly reports were submitted to KOFIH with anonymised statistics and activity summaries.

### Study population and variables

We included all individuals who received mobile clinic services during the study period. There was no pre-registration; all attendees were walk-in patients reached via community outreach, CSO referrals, or local announcements. All beneficiaries were foreign-born, regardless of their legal status or visa type and represented a diverse range of nationalities. Although we recorded 2737 clinical consultations, we based the analysis of coverage proportions on a deduplicated count of unique individuals (n = 2336), as identified through session-level records and provider assignments. We counted each individual only once, even if they received multiple services during a clinic session. We extracted the data from standardised encounter forms and mobilisation records maintained by partner agencies. Variables included nationality, service location, type of clinical services, and number and type of personnel. Demographic variables available for analysis included nationality and sex, which we extracted from standardised forms. We did not collect other attributes, such as age, legal status, or length of stay due to the programme’s service-oriented design. General services encompass non-clinical support, including general consultations, specialist care, chronic disease screenings, and health education. We did not record or analyse identifiable personal information.

### Data cleaning

Before the analysis, we manually cleaned all records using a standardised data cleaning process in Microsoft Excel, 2021 version (Microsoft Corporation, Redmond, Washington, USA). We excluded observations with missing or untraceable variables, such as visit date or service hours. Based on a combination of service location and provider assignment, we identified and subsequently removed duplicate entries. We performed logical cross-checks to ensure internal consistency between fields (*e.g.* alignment between service type and provider cadre). Notably, the data set used for the analysis was partially pre-processed at the point of submission of the routine report by Kyung Hee University Medical Center to KOFIH. Two researchers (Oh K and Kim Y) independently maintained an audit trail documenting all cleaning steps to ensure methodological transparency and reproducibility.

### Analytical approach

We used descriptive statistics to summarise demographics, clinic frequency by locality, and personnel deployment. We calculated the coverage proportion as the number of beneficiaries divided by the registered E-type visa holder population in each region (as of December 2024). We created graduated symbol maps using Microsoft Excel and Folium by Python, version 3.10.12 (Python Software Foundation, Wilmington, Delaware, USA) to visualise spatial variation, with a fixed-radius scale improving comparability. Generative artificial intelligence tools (*i.e.* ChatGPT, version GPT-4 (OpenAI, San Francisco, California, USA)) assisted with code refinement and automation; one researcher (Oh K) reviewed all outputs. We used a Kruskal-Wallis test to assess coverage differences across five regions (*i.e.* Seoul, Seoul Metropolitan Area excluding Seoul, Chungcheong, Jeolla, and Gyeongsang), followed by Dunn’s test with Bonferroni correction. We grouped the five regions based on administrative divisions and general patterns of migrant workers’ distribution in Korea. We excluded entries with missing or inconsistent data (*e.g.* service delivery hours, medical supplies).

### Visa classification and coverage denominator justification

In Korea, visa types correspond to foreigners’ primary purpose of stay, such as employment, study, family reunification, or investment. Among these, E-type visas are explicitly issued to individuals engaged in formal employment. These include low- and semi-skilled workers in manufacturing (E-9), agriculture (E-8), and shipbuilding (E-10), as well as professionals in specialised sectors (E-1–7). Collectively, E-type visa holders constitute the principal migrant workforce formally admitted under the Employment Permit System, and thus represent a key population for public health outreach. We used the number of registered E-type visa holders in each administrative region as of December 2024 as the denominator in calculating the coverage proportion. While the mobile clinic programme may have been open to all foreign individuals regardless of legal status or visa type, data on undocumented populations were not available at the regional level. We thus used E-type visa holders, who constitute the majority of Korea’s formal migrant workforce, as a conservative and policy-relevant proxy for population coverage estimates. This may result in an overestimation of actual coverage, but it provides a pragmatic and consistently measurable reference point for regional comparison. We obtained the data on E-type visa holders from the 2024 Korea Immigration Service Statistics, published by the Ministry of Justice [7]. These data provided disaggregated counts by administrative unit. The use of E-type visa data provides a consistent and conservative basis for evaluating the relative equity of service delivery across regions.

### Patient and public involvement

Although individual patients were not directly involved in the design or conduct of our study, CSOs representing migrant communities actively contributed to programme implementation. These partners played key roles in identifying local health needs, co-organising mobile clinic events, and supporting multilingual service access. We systematically incorporated the feedback from participating organisations and migrant beneficiaries to improve service protocols and logistical planning throughout the programme cycle.

### Ethical considerations

We based our analyses on secondary, anonymised data compiled during routine service operations, collecting no primary data. As we relied solely on anonymised secondary data and document-based review, institutional review board approval was not applicable. Mobile clinic operations were implemented under Article 23 of the Regional Public Health Act (Reporting of Health Check-up) and Article 27 of the Medical Service Act in Korea. We pre-coordinated and reported all activities to local health authorities in accordance with the Acts. We adhered to the ethical principles outlined in the Declaration of Helsinki.

## RESULTS

### Participant demographics and mobile clinic deployment

In total, 50 mobile clinic activities were carried out across 22 city- and district-level locations between May 2024 and March 2025 (**Table 2**). The highest concentration of activity occurred in Gyeonggi-do (42.0%), followed by Seoul (24.0%), Chungcheongnam-do (12.0%), and Jeollabuk-do (6.0%). At the city level, Danwon-gu in Ansan accounted for the most frequent service location (18.0%), followed by Gangdong-gu in Seoul (12.0%), and Dongnam-gu in Cheonan (10.0%). A total of 2336 migrant beneficiaries received the mobile clinic services. Of these, 58.6% were male and 41.4% female. The top five nationalities were Vietnamese (14.9%), Thai (10.7%), Filipino (9.6%), Chinese (8.9%), and Nepali (7.7%), representing diverse migrant groups from across Asia. A total of 1147 personnel were mobilised across all clinic sessions. The majority were volunteers (60.9%), followed by physicians (19.6%), nurses (9.7%), biomedical engineers (5.4%), and pharmacists (4.4%).

**Table 2:** Descriptive results of the mobile clinic operation in 2024

	n (%)
**Region**	9
Gyeonggi-do	21 (42.0)
Seoul Metropolitan City	12 (24.0)
Chungcheongnam-do	6 (12.0)
Jeollabuk-do	3 (6.0)
Incheon Metropolitan City	2 (4.0)
Daegu Metropolitan City	2 (4.0)
Gwangju Metropolitan City	2 (4.0)
Gyeongsangnam-do	1 (2.0)
Jeollanam-do	1 (2.0)
City*	22
Danwon-gu (Ansan)	9 (18.0)
Gangdong-gu (Seoul)	6 (12.0)
Dongnam-gu (Cheonan)	5 (10.0)
Hwaseong	4 (8.0)
Namdong-gu (Incheon)	3 (6.0)
Seongdong-gu (Seoul)	2 (4.0)
Gwangsan-gu (Gwangju)	2 (4.0)
Dongducheon	2 (4.0)
Dalseo-gu (Daegu)	2 (4.0)
Yongsan-gu (Seoul)	2 (4.0)
Yangju	2 (4.0)
Masan-Happo (Changwon)	1 (2.0)
Jinan	1 (2.0)
Deokjin-gu (Jeonju)	1 (2.0)
Yangcheon-gu (Seoul)	1 (2.0)
Iksan	1 (2.0)
Goheung	1 (2.0)
Pocheon	1 (2.0)
Pyeongtaek	1 (2.0)
Taean	1 (2.0)
Jungnang-gu (Seoul)	1 (2.0)
Gwangju (Gyeonggi-do)	1 (2.0)
**Beneficiary gender**	2336
Male	1370 (58.6)
Female	966 (41.4)
**Beneficiary nationality**	2336
Vietnam	349 (14.9)
Thailand	250 (10.7)
Philippines	225 (9.6)
China	207 (8.9)
Nepal	179 (7.7)
Mongolia	162 (6.9)
Uzbekistan	123 (5.3)
Bangladesh	113 (4.8)
Cambodia	107 (4.6)
Sri Lanka	63 (2.7)
Russia	62 (2.7)
Pakistan	55 (2.4)
India	46 (2.0)
Indonesia	42 (1.8)
Myanmar (Burma)	30 (1.3)
Kazakhstan	28 (1.2)
Lao PDR	14 (0.6)
Others	281 (12.0)
**Mobilised personnel**	1147
Physician	225 (19.6)
Nurse	111 (9.7)
Biomedical Engineer	62 (5.4)
Pharmacist	50 (4.4)
Volunteer	699 (60.9)
Clinics	2517
Internal Medicine	1312 (47.9)
Traditional Korean Medicine	250 (9.1)
Orthopaedics	241 (8.8)
Family medicine	209 (7.6)
Surgery	46 1.7()
Dermatology	39 (1.4)
Urology	30 (1.1)
Physical medicine and rehabilitation	23 (0.8)
Clinical Pathology	9 (0.3)
Neurology	0 (0.0)
General services†	578 (21.1)

*While the variable was classified at the city level, we subdivided Seoul, the capital, into districts due to its population structure.

†General services include health promotion, health education, and counselling activities.

### Geographic coverage disparities and spatial analysis

**Figure 2:**
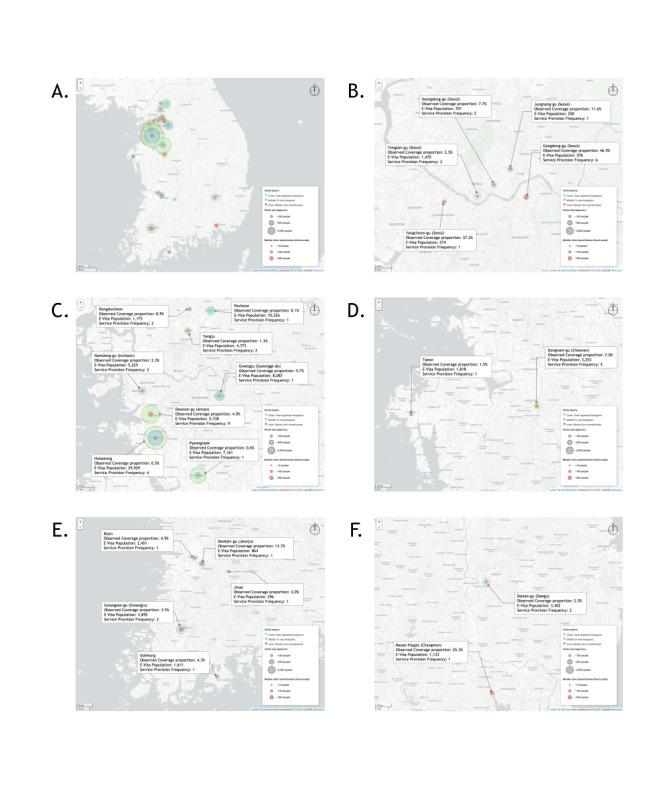
Geographic coverage proportion of the mobile clinic programme across 22 administrative regions in Korea, 2024. Concentric circles represent (I) total registered foreign residents, (II) E-type visa holders, and (III) mobile clinic beneficiaries. **Panel A.** National overview: The mobile clinic activities in 2024 exhibited significant regional disparities, with a particular concentration in the Seoul Metropolitan Area, including Seoul. **Panel B.** Seoul: Gangdong-gu shows the highest coverage (46.9%) with six deployments, while other districts such as Yangcheon-gu (37.2%) and Seongdong-gu (7.7%) show moderate reach. **Panel C.** Seoul Metropolitan Area (excluding Seoul): Hwaseong and Pyeongtaek, despite large E-type visa populations, had coverage below 1%, contrasting with Ansan’s 4.0% from nine deployments. **Panel D.** Chungcheong Area: Dongnam-gu in Cheonan received five visits (7.0% coverage), while Taean had minimal service (1.5%). **Panel E.** Jeolla Area: Deokjin-gu in Jeonju had 13.7% coverage from a single visit, with moderate outreach in Gwangsan-gu in Gwangju, Iksan, and Goheung. **Panel F.** Gyeongsang Area: Masan-Happo-gu in Changwon reached 25.3% coverage with one visit, contrasting with limited service in Dalseo-gu in Daegu, 2.5%).

To assess geographic equity in access to mobile healthcare services, we conducted a coverage proportion analysis, comparing the number of mobile clinic beneficiaries to the registered population of foreign residents across 22 city- and district-level locations (**Figure 2**, Panels A–F). We drew beneficiary circles representing total registered foreigners; E-type visa holders, and mobile-clinic beneficiaries using a fixed visual scale (~10, 100, and 300 persons) to enhance comparability and to highlight disparities in service outreach relative to the size of the target population. The spatial distribution revealed marked inequities in mobile clinic coverage. Some districts in Seoul demonstrated relatively high coverage proportions, including Gangdong-gu (46.9%), Yangcheon-gu (37.2%), and Seongdong-gu (7.7%). Moderate levels were seen in Dongnam-gu in Cheonan (7.0%) and Deokjin-gu in Jeonju (13.7%). In contrast, districts with large E-type visa populations, such as Hwaseong (0.5%), Pyeongtaek (0.6%), and Gwangju in Gyeonggi-do (0.7%), exhibited low coverage. Although Gyeonggi-do and Seoul had the largest E-type visa populations, clinic coverage did not consistently correlate with population size.

**Figure 3:**
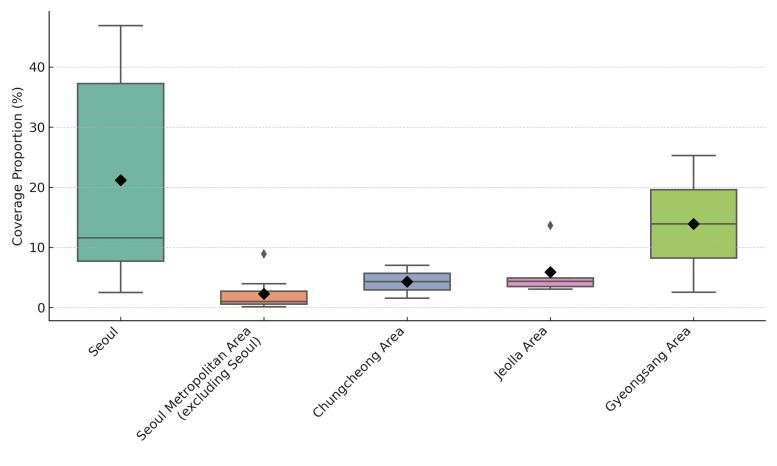
Coverage proportion of mobile clinic services by region (n = 22). The black diamond indicates the mean coverage for each region, while the grey circle represents statistical outliers, defined as values >1.5 times the interquartile range. Interpretation should be made with caution in regions with small samples (*e.g. *Chungcheong Area and Gyeongsang Area, each n = 2). Outlier regions may reflect targeted deployments or exceptionally strong local partnerships.

To account for Seoul’s dense and diverse population distribution, we disaggregated the city into its constituent districts, allowing for a more granular visualisation of service reach. The differences in coverage proportions across the five regional clusters were not significant (H = 9.470; df = 4; *P* = 0.0503; 95% confidence interval = 4.37–17.51). However, a *post-hoc* analysis revealed a statistically significant difference between Seoul and the Seoul Metropolitan Area excluding Seoul (z = 2.46; *P* = 0.0410; 95% confidence interval = 0.91–4.83), indicating that coverage was significantly higher in Seoul districts. No other pairwise comparisons remained significant after adjustment. Boxplot analysis revealed that Seoul had the highest median coverage, followed by the Jeolla Area and the Chungcheong Area. In contrast, the Seoul Metropolitan Area, excluding Seoul and the Gyeongsang Area, showed lower and more variable coverage (**Figure 3**). These findings collectively point to the need for data-informed prioritisation in mobile service planning and more systematic integration of community actors into outreach strategies.

### Health service delivery and civil society involvement

Among the 1147 personnel mobilised throughout the programme, 699 (60.9%) were volunteers, while the remaining 448 (39.1%) were medical professionals, including 225 (19.6%) physicians, 111 (9.7%) nurses, 62 (5.4%) biomedical engineers, and 50 (4.4%) pharmacists. In terms of clinical services, 2737 individual consultations were recorded, reflecting repeat visits and multi-speciality care provided to some individuals. The most delivered services were internal medicine (47.9%), traditional Korean medicine (9.1%), and orthopaedics (8.8%). Other specialities, such as family medicine (7.6%) and dermatology (1.4%), were also represented, while certain specialities, such as neurology, were not provided during the research period. General services, which accounted for 21.1% of all consultations, included non-speciality care, such as health promotion activities, health counselling, chronic disease screenings, and basic health education. The mobile clinics’ implementation heavily relied on collaboration with CSOs, including faith-based and volunteer-led groups, which co-hosted or facilitated nearly all deployments. A total of 18 organisations participated in implementation across 50 events (Figure S1 in the **Online Supplementary Document**). These organisations not only co-hosted clinics but also provided critical functions such as outreach and mobilisation, interpretation and cultural mediation, and logistical support. Their involvement enabled linguistically and culturally appropriate service delivery in areas with limited institutional infrastructure.

## DISCUSSION

### Mobile clinics and equity in migrant health

Mobile clinics have increasingly been recognised as an effective strategy to deliver health services to underserved, marginalised and hard-to-reach populations, including migrant workers, rural residents, and homeless communities. Evidence from the USA, Southeast Asia, and Europe demonstrates the potential of mobile units to bridge access gaps by addressing financial, linguistic, and geographical barriers [18–20]. In Korea, however, peer-reviewed studies on mobile clinics targeting foreign residents and migrant workers remain scarce. Existing studies tend to focus on single-centre outreach efforts or qualitative descriptions of needs and barriers, rather than systematically analysing coverage levels, regional disparities, or the operational structure of service delivery [3,8,9].

### Key findings and novel contributions

We offer novel and practical insights into KOFIH’s 2024 mobile clinic programme, conducted in collaboration with Kyung Hee University Medical Center and CSOs. We provide a multi-layered assessment of participant characteristics and service volume, regional coverage disparities, and CSO-led service delivery. The use of a coverage proportion metric offers a quantitative lens to assess equity in access and visualise spatial variation. This is one of the first Korean studies to examine the relationship between migrant population size and actual service utilisation across multiple regions while also highlighting the role of faith-based and community-led organisations in enabling access. We expand prior research on structural barriers by illustrating implementation strategies that mitigate such obstacles through decentralised and culturally adapted delivery [6,21,22]. Our results revealed three central insights. First, most beneficiaries were male migrant workers from Southeast Asia, particularly Vietnam, Thailand, and the Philippines, reflecting Korea’s dependence on low-skilled foreign labour in industrial and agricultural sectors [7,8]. Clinics located near workplaces and dormitories were especially effective in reaching these populations. However, the gender imbalance suggests a need for more targeted outreach to female migrants, who may face unique access barriers such as caregiving responsibilities or fear of deportation [9,23]. Second, geospatial analysis revealed marked disparities in coverage proportions across regions, even in areas with large migrant populations. To account for Seoul’s dense and diverse population distribution, we disaggregated the city into its constituent districts, allowing for a more granular visualisation of service reach. This supports global evidence that ‘proximity does not equal accessibility’ [24], particularly for socially marginalised groups. The Kruskal-Wallis test was not significant, although the result was close to the 0.05 threshold. *Post-hoc* Dunn’s test identified a statistically significant difference between Seoul and its surrounding metropolitan area, suggesting meaningful variation not fully captured by the omnibus test. Notably, the interquartile ranges in Seoul and Gyeongsang Area were extensive, indicating internal heterogeneity in service coverage within these regions. Seoul’s higher coverage may reflect stronger civil society infrastructure, established partnerships, and local prioritisation, while lower coverage in the Seoul Metropolitan Area (excluding Seoul) and Gyeongsang Area points to weaker institutional support. These findings underscore the importance of regionally tailored outreach, data-informed planning, and CSO collaboration. Third, the implementation model demonstrated the strength of decentralised, CSO-driven service delivery. Across 50 deployments, involving 18 partners, including faith-based groups (*e.g.* Christian and Buddhist), non-governmental organisations, health institutions, and migrant support centres, culturally and linguistically responsive care was enabled. This aligns with global community-based models in which social capital and co-produced service design improved health outcomes and satisfaction [25]. The integration of both conventional and traditional medicine further enhanced accessibility. These findings provide a spatially grounded, equity-oriented model for delivering migrant health services. The high level of community engagement, particularly through faith-based networks and CSOs, highlights the critical role of grassroots as a backbone for providing healthcare to medically underserved populations, in particular, migrant workers. By applying a coverage proportion metric and visual mapping across 22 sites, we quantified operational and geographic disparities, reinforcing the essential role of CSOs. We also contributed a replicable framework for monitoring health access equity using routine programme data.

### Global health policy implications

Although we focussed on the Korean context, the findings may have broader implications for global migrant health equity. The use of mobile clinics as a flexible and community-integrated strategy can be adapted to various contexts, particularly in high-income countries with significant migrant workers. The evidence that geographic proximity alone does not guarantee equitable access to care highlights the critical need for community partnerships, multilingual service navigation, and data-informed resource allocation in migrant health programmes across the globe. Furthermore, as widely recognised, the integration of CSOs as active stakeholders in service design and delivery offers a scalable model to bridge access gaps in health systems facing demographic shifts and labour migration pressures [26–29].

### National policy implications and future directions

We offer several actionable insights for public health planning. First, achieving equity in service coverage requires more than just geographic outreach; it demands data-informed site selection and coordination with local actors who understand the dynamics of migrant communities. Second, standardised coverage metrics can support strategic planning and accountability, especially when disaggregated at the city or district level. Third, civil society should be embedded not merely as delivery agents, but as equal partners in programme design and governance. To institutionalise the enabling role of CSOs, national migrant health policy frameworks could formally recognise community-based organisations as core delivery partners, such as through their inclusion in national or subnational health planning and governance structures. Such integration would not only sustain outreach capacity but also enhance cultural responsiveness and trust among migrant populations. Public financing systems should evolve to support these roles through formalised mechanisms.

As Korea’s foreign-born population continues to expand, mobile clinics can serve as scalable and inclusive platforms for service delivery. However, scalability must be matched by structural reform. Future models should move beyond visa-type segmentation to include a broader range of migrant populations, adopt multilingual patient-tracking tools, and integrate civil society actors through co-governance structures that ensure sustainability and responsiveness.

### Limitations and recommendations

This study has several limitations. First, we calculated the coverage proportions using the number of registered E-type visa holders per region as of December 2024. This approach was based on three considerations: E-type visa holders are the programme’s primary policy target; they are systematically recorded, allowing reliable regional estimates; and data on undocumented or short-term migrants were unavailable. While visa status was not a criterion for receiving services, and the exact distribution of beneficiaries by visa type, either documented or undocumented, was not captured, this denominator may have modestly overestimated coverage. Additionally, using a single time point (December 2024) does not reflect potential fluctuations in the migrant population over the programme period. Nevertheless, this approach provides a conservative and operationally consistent basis for assessing regional equity [7]. Second, the aggregated, cross-sectional data limited our ability to analyse individual-level outcomes, repeat utilisation, or continuity of care. Without patient identifiers or follow-up mechanisms, it is impossible to evaluate effectiveness and satisfaction. Future programmes should consider anonymised tracking systems. Third, we selected service locations based on CSO requests and logistical feasibility, not through randomisation or systematic criteria. This may introduce selection bias and limit generalisability. Structured site selection, using population density, unmet needs, or vulnerability indices, is recommended. Fourth, we lacked data on social determinants, including housing, employment, legal status, length of stay, health literacy, and age. These are essential for understanding access barriers and utilisation disparities. Additionally, we were unable to assess differences in regional health needs or disease prevalence, which may have contributed to variations in service coverage across locations. Future monitoring should incorporate such variables, including age-disaggregated analyses. Finally, as this was a descriptive study without a control group, we could not draw causal inferences. While the coverage proportion metric offers a valuable lens on equity, more robust designs, such as quasi-experimental or mixed-method studies, are needed to evaluate programme effectiveness, cost-effectiveness, and user experience. Despite these limitations, our study demonstrates a practical framework for assessing migrant health equity using routine data and geospatial tools. Future efforts should prioritise integrated data systems, inclusive targeting, and participatory governance to enhance health service delivery for migrant populations.

## CONCLUSIONS

We provide one of the first nationwide analyses in Korea to examine the relationship between the size of the migrant worker population and actual utilisation of mobile clinic services across five regions. Despite operating in areas with high concentrations of migrant workers, the programme demonstrated substantial variability in coverage, indicating that geographic proximity alone does not ensure equitable access. The integration of geospatial tools with routine programme data presents a replicable framework for monitoring service equity. To strengthen primary healthcare access for all migrants, including both documented and undocumented populations, mobile health strategies must be guided by disaggregated data, locally adapted service models, and sustained engagement with CSOs. Policymakers, health authorities, and public entities related to migrant workers should institutionalise participatory site selection, multilingual navigation support, and equity-focused monitoring systems. Embedding these approaches into planning for mobile clinic operations and further national health planning can transform mobile clinics from temporary outreach tools into sustainable components of an inclusive health system.

## Additional material


Online Supplementary Document
