# Supplementary material for: Coverage disparities in mobile health services for migrant workers in Korea: a spatial equity analysis
Source: J Glob Health. 2025 Oct 17;15:04300. doi: 10.7189/jogh.15.04300 (PMC12532446; doi:10.7189/jogh.15.04300)
Supplement: Online Supplementary Document [file jogh-15-04300-s001.pdf]

Supplement to: Oh K, Kim Y, Park W, Cheon J. Coverage disparities in mobile health services for migrant workers in Korea: a spatial equity analysis. J Glob Health. 2025;15:04300.

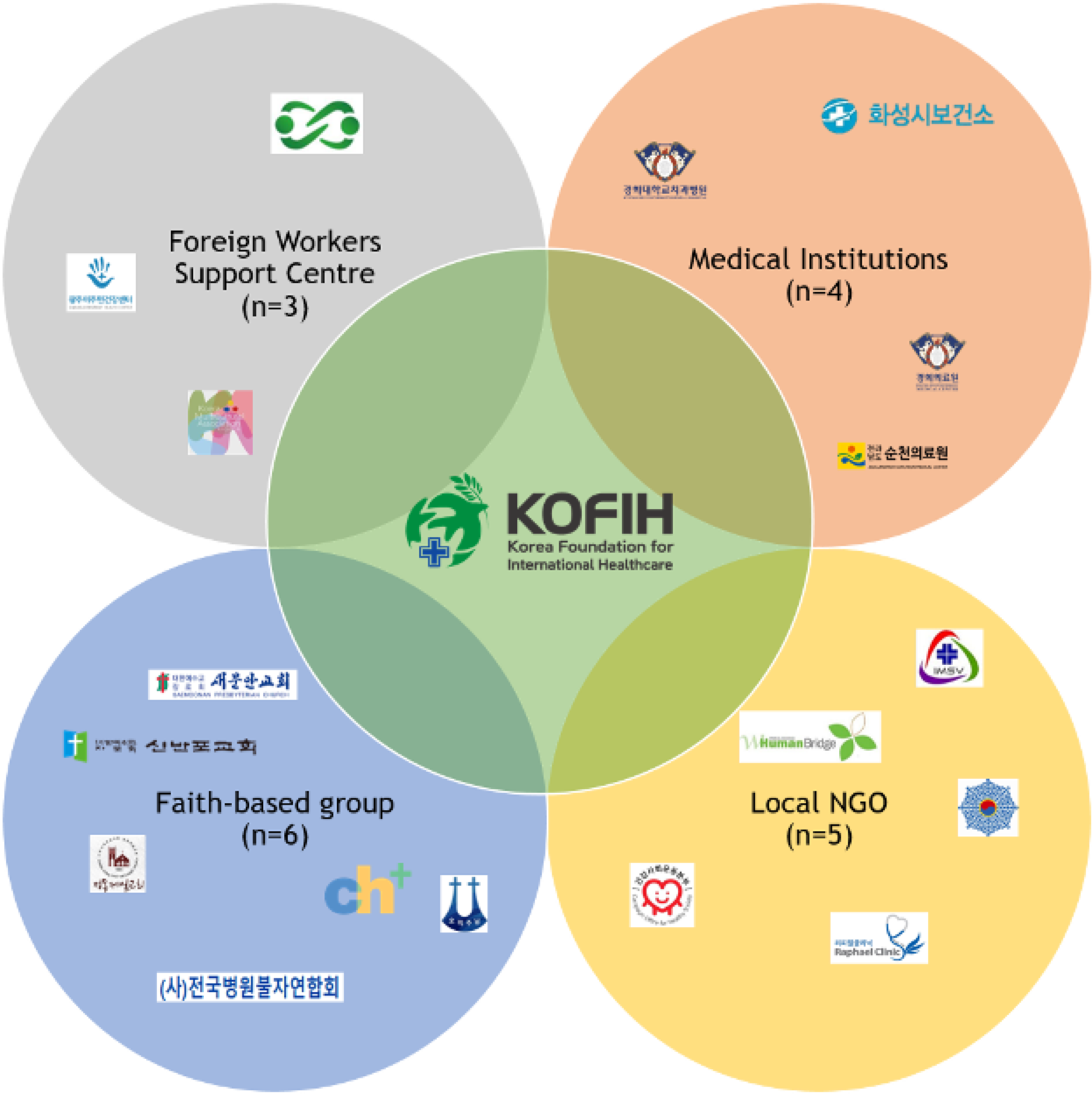

Figure S1. Classification of partner organisations participating in the mobile clinic programme (n = 18).
